# Robot-assisted laparoscopic debulking surgery for recurrent adult granulosa cell tumors

**DOI:** 10.1016/j.gore.2021.100783

**Published:** 2021-05-07

**Authors:** Jolijn W. Groeneweg, Joline F. Roze, Wouter B. Veldhuis, Jelle P. Ruurda, Cornelis G. Gerestein, Ronald P. Zweemer

**Affiliations:** a Department of Gynecologic Oncology, University Medical Center Utrecht, Utrecht University, Utrecht, The Netherlands; b Department of Radiology, University Medical Center Utrecht, Utrecht University, Utrecht, The Netherlands; c Department of Surgery, University Medical Center Utrecht, Utrecht University, Utrecht, The Netherlands

**Keywords:** Granulosa cell tumor, Robot-assisted laparoscopy, Debulking, Cytoreductive surgery, Recurrence

## Abstract

Despite an often early diagnosis and effective initial surgical management, one third of adult granulosa cell tumors (aGCTs) eventually, and often repeatedly, recurs. Debulking surgery remains the preferred treatment modality for recurrent aGCT, although the risk of intraoperative complications increases with repeated laparotomy. Minimally invasive surgery may limit the risk of complications. We aim to share our initial experience with robotic debulking surgery for recurrent aGCT. Clinical and surgical data of patients with recurrent aGCT who underwent robotic cytoreductive surgery over a three-year period at a tertiary referral center were retrospectively collected and analyzed. Between 2017 and 2020, three patients underwent robotic debulking surgery for recurrent aGCT at our institution. Complete cytoreduction was achieved in all patients. No intraoperative or postoperative complications were reported. This small pilot series at a single academic institution suggests that robot-assisted laparoscopy may be feasible and safe in selected patients with recurrent aGCT. A minimally invasive approach could reduce the complexity of successive surgeries for aGCT relapse.

## Highlights

• In this pilot study, the use of robotic debulking surgery for recurrent granulosa cell tumors was retrospectively analyzed.
• Robot-assisted laparoscopic debulking surgery resulted in complete cytoreduction in all patients.
• No perioperative complications were reported following robotic cytoreductive surgery.
• A minimally invasive approach could reduce the complexity of successive surgeries for granulosa cell tumor relapse.

## 1 Introduction

Granulosa cell tumors (GCTs) of the ovary represent a rare subtype of ovarian cancer, belonging to the subgroup of sex cord-stromal cell tumors and accounting for approximately 3–5% of all ovarian malignancies. The vast majority of GCTs is of the adult type (aGCT), while 5% is of the juvenile type. Although aGCTs are generally described as tumors with an indolent behavior that are often diagnosed at an early stage with a favorable prognosis, one third of aGCT patients eventually develops a disease relapse leading to death in 50–80% of recurrences (Sun et al., 2012, Bryk et al., 2016, Farkkila et al., 2017). Surgery is the mainstay of treatment for both primary and recurrent aGCT (Colombo et al., 2007). Only limited effectiveness of alternative treatment modalities such as chemotherapy and hormonal treatment has been described (Van Meurs et al., 2014, Van Meurs et al., 2015). Despite optimal surgical debulking of relapsed aGCT, multiple recurrences are commonly seen. Repeated debulking surgery is therefore often necessary in this setting, with increased risk of intraoperative complications with every laparotomy.

In recent years, minimally invasive surgery is increasingly used in the treatment of ovarian cancer. Advantages of this surgical approach over laparotomy have been widely described and include smaller incisions, reduced blood loss, improved intraoperative visualization, shorter hospitalization, faster recovery and a lower risk of formation of adhesions (Levrant et al., 1997, Polymeneas et al., 2001). Laparoscopy and robot-assisted laparoscopy were found to be feasible and safe surgical routes in selected patients with primary or recurrent epithelial ovarian cancer, in terms of surgical and oncologic outcomes (Tantitamit and Lee, 2018, Cardenas-Goicoechea et al., 2019, Eriksson et al., 2017).

The advantages of a minimally invasive surgical route could be particularly meaningful for the management of aGCT, when multiple surgeries may be needed for relapsed disease. In the primary treatment of aGCT, laparoscopy was shown to be an accurate approach for both initial surgery and re-staging, with comparable oncologic outcomes when compared with laparotomy (Peiretti et al., 2020, Bergamini et al., 2018). In addition, a recent case report showed the use of laparoscopy for tertiary cytoreductive surgery in recurrent aGCT (Pineda et al., 2020). The use of robot-assisted laparoscopy for the surgical treatment of aGCT has not yet been reported. With this small series from a single institution, we aim to share our initial, positive experience with robotic surgery for the treatment of recurrent aGCT.

## 2 Materials and methods

Patients who underwent robotic cytoreductive surgery for a recurrent aGCT in our institution between June 2017 and July 2020 were retrospectively analyzed. All patients had given written informed consent for their data to be used for study purposes, with approval of our institutional review board (METC 17–868).

All surgeries were performed by two gynecologic oncologists (R.P.Z. and C.G.G.) experienced in robot-assisted laparoscopy, in some cases accompanied by a gastro-intestinal surgeon specialized in robotic surgery (J.P.R.). The da Vinci Surgical System (Intuitive Surgical Inc., Sunnyvale, CA, USA) was used, the da Vinci Si for the first case and the da Vinci Xi for the second and third case. For all procedures, patients were placed in lithotomy position. Following a small incision just below or just above the umbilicus, depending on the upper or lower intra-abdominal locations of the intended procedure, a pneumoperitoneum of 24 mmHg was created using a Veress needle. The camera port was placed, followed by placement of three robotic ports and one laparoscopic port for the assistant, all in one line at the level of the camera port. In one case, an additional suprapubic assistant port was placed. After lowering the intra-abdominal pressure to 14 mmHg and routine inspection of the peritoneal cavity, the da Vinci robot was docked and surgical instruments were introduced with the patient in 28° Trendelenburg position.

Patient characteristics and operative outcomes were collected from medical records. The clinical parameters collected for each patient included: age at time of surgery, body mass index (BMI), history of smoking, American Society of Anesthesiologists (ASA) classification, previous abdominal surgery, initial aGCT stage, months after initial diagnosis, previous treatment for aGCT, recurrence number, inhibin B level, tumor locations as seen on CT or MRI scan, number of lesions and size of largest lesion. The studied perioperative characteristics included the performed surgical procedure, operative (cutting) time, estimated blood loss, need for blood transfusion, conversion to laparotomy, cytoreduction status, length of hospital stay, complications and readmission. The length of hospital stay was counted from the day of surgery. Complications were registered using the Clavien-Dindo classification of postoperative complications.

## 3 Results

**Fig. 1:**
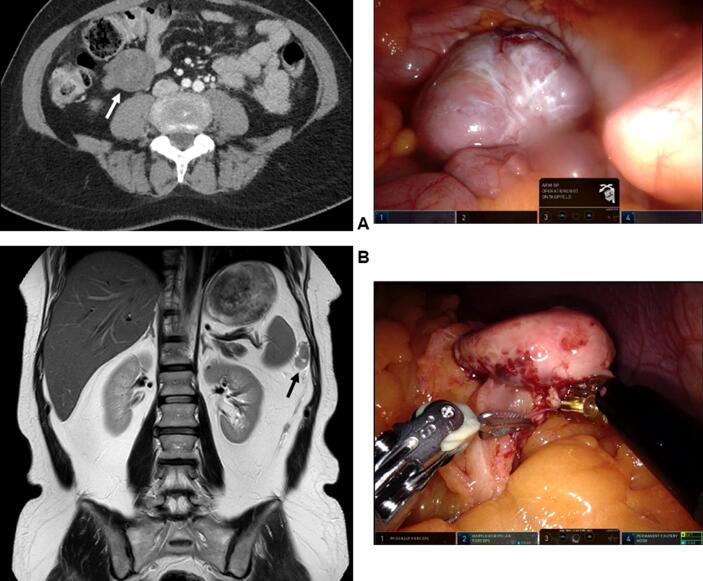
Imaging of recurrent aGCT lesions and correlating image of the robotic surgery. A: Patient 2. The deposit on the mesocolon is shown on CT imaging (left) and during surgery (right). B: Patient 3. A paracolic peritoneal deposit is shown on MRI imaging (left) and during surgery (right).

Between June 2017 and July 2020, ten patients underwent debulking surgery for recurrent aGCT at our institution, of which three patients were treated by robot-assisted laparoscopy. They were estimated to be good candidates for robotic debulking surgery based on preoperative CT or MRI findings. All three patients were treated for their first recurrence. The age at time of surgery ranged from 51 to 74 years (Table 1). The first patient had a unifocal recurrence in the pelvis, for which a robot-assisted laparoscopic resection of the tumor was performed (Table 2). The other two patients were found to have multifocal peritoneal disease. In the second patient, preoperative CT imaging showed two peritoneal deposits on the spleen and one deposit on the mesocolon. In the third patient, a preoperative MRI scan showed peritoneal deposits in Douglas and left paracolic gutter as well as in Morisońs pouch. Their robotic cytoreductive surgery included a hysterectomy with unilateral salpingo-oophorectomy and selective peritonectomy to remove the peritoneal deposits (Fig. 1). Macroscopically complete cytoreduction was achieved in all three patients. The operative time, defined as the time between the first incision and final closure, was 99 min for the first case, 231 min for the second case and 162 min for the third case. The first patient had a hospital stay of nine days due to social circumstances unrelated to her surgery, and the second and third patient had a hospital stay of three days. No intraoperative or postoperative complications occurred.

**Table 1:** Clinical characteristics of study patients.

	Patient 1	Patient 2	Patient 3
**Age (years)**	74	51	58
**BMI (kg/m^2^)**	36	23	25
**Smoking**	No	No	No
**ASA classification**	3	2	2
**Previous abdominal surgery**	Vaginal hysterectomy with BSO	Laparoscopic USO	Laparoscopic USO Two caesarean sections
**Initial aGCT stage**	IA	IC1	IC1
**Months after initial diagnosis**	73	65	78
**Previous treatment for aGCT**	Surgery	Surgery	Surgery
**Recurrence #**	1	1	1
**Inhibin B level (ng/L)**	86	129	144
**Tumor locations**	Pelvis left	Peritoneum of spleen, mesocolon	Peritoneal deposits
**Number of lesions**	1	>5	>5
**Size of largest lesion**	89 mm	43 mm	30 mm

ASA: American Society of Anesthesiologists; BSO: bilateral salpingo-oophorectomy; USO: unilateral salpingo-oophorectomy.

**Table 2:** Operative outcomes.

	Patient 1	Patient 2	Patient 3
**Procedure**	Resection of pelvic tumor	Hysterectomy, USO, selective peritonectomy	Hysterectomy, USO, selective peritonectomy, partial omentectomy
**Operative time**	99 min	231 min	162 min
**Estimated blood loss**	100 ml	400 ml	50 ml
**Blood transfusion**	No	No	No
**Conversion to laparotomy**	No	No	No
**Cytoreduction status**	Complete	Complete	Complete
**Length of hospital stay**	9 days	3 days	3 days
**Complications**	None	None	None
**Readmission**	No	No	No

USO: unilateral salpingo-oophorectomy.

The first patient had no signs of disease at her most recent follow-up, three years after the robotic debulking surgery. The second patient had her most recent follow-up eight months after her surgery, and was then found to be in good clinical condition but with a mildly increased inhibin B level (98 ng/L to 133 ng/L). The third patient has only had a three-month follow-up since her surgery, when there were no signs of disease despite a continued elevated inhibin B (144 ng/L before surgery and 133 ng/L after surgery).

## 4 Discussion

With this small pilot series, we describe the use of robot-assisted laparoscopy in recurrent aGCT. In all three cases, a first unifocal or multifocal recurrence of aGCT could be completely removed by robotic cytoreductive surgery. No intraoperative or postoperative complications occurred. These findings suggest that in selected patients, robot-assisted laparoscopy may be a safe and effective surgical approach in recurrent aGCT.

Only limited evidence exists regarding the role of laparoscopy in the surgical treatment of aGCT. One retrospective study found no differences in oncologic outcomes between laparoscopy and laparotomy in the initial treatment of patients with stage I aGCT, after a median follow-up of 81 months (Bergamini et al., 2018). A second retrospective analysis demonstrated the feasibility of laparoscopic re-staging in patients with incompletely staged aGCT (Peiretti et al., 2020). Finally, a recent video article showed the successful use of laparoscopy for cytoreductive surgery in aGCT recurrence (Pineda et al., 2020). In epithelial ovarian cancer, the role of minimally invasive surgery has been assessed by multiple observational studies. Available evidence on the use of laparoscopic or robotic staging for early stage ovarian cancer suggests that minimally invasive staging procedures are feasible and do not compromise oncologic outcomes (Bellia et al., 2016, Ghezzi et al., 2012). In patients with advanced stage epithelial ovarian cancer, interval cytoreductive surgery by laparoscopy or robot-assisted laparoscopy was found to be adequate in selected patients (Gueli Alletti et al., 2016, Melamed et al., 2017, Ackroyd et al., 2018, Fagotti et al., 2019). The MISSION trial, a phase II multicenter study, reported the feasibility and safety of minimally invasive interval debulking surgery in patients with a clinically complete response to neoadjuvant chemotherapy (Gueli Alletti et al., 2016). Furthermore, the International MISSION study demonstrated the benefits of a minimally invasive approach when interval surgery is limited to low-complexity standard cytoreductive procedures (Fagotti et al., 2019). An international, randomized, multicenter phase III trial will be conducted to compare minimally invasive interval debulking surgery with laparotomy in patients who had a complete or partial response to neoadjuvant chemotherapy (Nitecki et al., 2020). In addition to its use for primary treatment, other studies have shown favorable perioperative outcomes and similar survival rates when using a minimally invasive approach for secondary cytoreductive surgery in recurrent epithelial ovarian cancer (Eriksson et al., 2017, Gallotta et al., 2018, Magrina et al., 2013). In line with these previous studies in both aGCT and epithelial ovarian cancer, our series of three patients with recurrent aGCT surgically treated by robot-assisted laparoscopy suggests that this approach is safe and adequate in selected cases. In these previous reports as well as in our study, patients were selected for minimally invasive surgery when preoperative evaluation showed a limited burden of disease in areas deemed accessible by (robot-assisted) laparoscopy.

Our findings are particularly relevant in aGCT, where surgery represents the mainstay of treatment for both primary and recurrent disease. Minimally invasive surgery offers advantages such as less blood loss, shorter hospitalization and faster recovery when compared with open surgery. When repeated abdominal surgery is needed, which is not uncommon in the setting of a recurrent aGCT, a minimally invasive surgical route could potentially reduce the risk of intraoperative complications with subsequent surgeries. Prior laparoscopy was previously shown to significantly reduce the formation of anterior abdominal wall adhesions when compared with prior laparotomy (Levrant et al., 1997, Polymeneas et al., 2001, Burns et al., 2013). In addition, significantly fewer unfavorable incidents during subsequent laparoscopy were reported in patients who had previous laparoscopic colorectal cancer surgery, compared with patients who had previous open surgery (Di Fabio et al., 2015).

The present study is the first to report the use of robot-assisted laparoscopy for recurrent aGCT, as demonstrated by an experienced surgical team. The importance of experience in robotic surgery and its influence on oncologic outcomes in cervical cancer has recently been reported by our group (Baeten et al., 2020). However, important limitations of the current study include the small size of our series, its retrospective nature and relatively short follow-up. Further collaborative research is warranted to confirm our findings supporting the use of robot-assisted laparoscopy in selected patients with recurrent aGCT.

In conclusion, debulking surgery using robot-assisted laparoscopy may be feasible and safe in selected patients with recurrent aGCT. A minimally invasive approach could reduce the complexity of successive surgeries for aGCT relapse.

## CRediT authorship contribution statement

**Jolijn W. Groeneweg:** Conceptualization, Investigation, Formal analysis, Visualization, Writing - original draft, Writing - review & editing. **Joline F. Roze:** Conceptualization, Investigation, Writing - review & editing. **Wouter B. Veldhuis:** Investigation, Visualization, Writing - review & editing. **Jelle P. Ruurda:** Writing - review & editing. **Cornelis G. Gerestein:** Conceptualization, Supervision, Writing - review & editing. **Ronald P. Zweemer:** Conceptualization, Supervision, Funding acquisition, Writing - review & editing.

## Declaration of Competing Interest

The authors declare that they have no known competing financial interests or personal relationships that could have appeared to influence the work reported in this paper.
